# Pilot study of a community pharmacist led program to treat hepatitis C virus among people who inject drugs

**DOI:** 10.1016/j.dadr.2023.100213

**Published:** 2023-12-23

**Authors:** J.I. Tsui, A.J. Gojic, K.A. Pierce, E.L. Tung, N.C. Connolly, A.C. Radick, R.R. Hunt, R. Sandvold, K. Taber, M. Ninburg, R.H. Kubiniec, J.D. Scott, R.N. Hansen, J.D. Stekler, E.J. Austin, E.C. Williams, S.N. Glick

**Affiliations:** a Department of Medicine, University of Washington School of Medicine, Division of General Internal Medicine University of Washington, Seattle, WA, United States; b Kelley-Ross Pharmacy Group, Seattle, WA, United States; c Department of Pharmacy, University of Washington, Seattle, WA, United States; d Des Moines University College of Osteopathic Medicine, Des Moines, IA, United States; e Hepatitis Education Project, Seattle, WA, United States; f Evergreen Treatment Services, Seattle, WA, United States; g Department of Health Systems and Population Health, University of Washington, Seattle, WA, United States; h Department of Medicine, Division of Allergy and Infectious Diseases, University of Washington, Seattle WA, United States; i Seattle-Denver Center of Innovation for Veteran-Centered and Value-Driven Care, Health Services Research & Development, VA Puget Sound, Seattle WA, United States; j HIV/STI/HCV Program, Public Health - Seattle & King County, Seattle WA, United States

**Keywords:** Hepatitis C virus, Antiviral drugs, Substance addiction, Injection drug use

## Abstract

**Background:**

People who inject drugs (PWID) are a key population for treatment with direct-acting antiviral medications (DAAs) to eliminate hepatitis C virus (HCV). We developed a Pharmacist, Physician, and Patient Navigator Collaborative Care Model (PPP-CCM) for delivery of HCV treatment; this study describes clinical outcomes related to HCV treatment (initial evaluation, treatment initiation, completion, and cure), as well as patient satisfaction.

**Methods:**

We conducted a single-arm prospective pilot study of adult PWID living with HCV. Participants completed baseline and six-month follow-up surveys, and treatment and outcomes were abstracted from electronic health records. Primary outcome was linkage to pharmacist for HCV evaluation; secondary outcomes included DAA initiation, completion, and cure, as well as patient-reported satisfaction.

**Results:**

Of the 40 PWID enrolled, mean age was 43.6 years, 12 (30 %) were female, 20 (50 %) were non-white, and 15 (38 %) were unhoused. Thirty-eight (95 %) were successfully linked to the pharmacist for initial evaluation. Of those, 21/38 (55 %) initiated DAAs, and 16/21 (76 %) completed treatment. Among those completing treatment who had viral load data to document whether they achieved “sustained virologic response", i.e. cure, 10/11 (91 %) were found to be cured. There was high satisfaction with 100 % responding “agree or strongly agree” that they had a positive experience with the pharmacist.

**Conclusion:**

Nearly all participants in this pilot were successfully linked to the pharmacist for evaluation, and more than half were started on DAAs; results provide preliminary evidence of feasibility of pharmacist-led models of HCV treatment for PWID.

**Clinicaltrials.gov registration number:**

NCT04698629.

## Highlights

• A collaborative, pharmacist-led model treated hep C among people who use drugs (PWID).
• Over half started hep C meds; majority of those who completed meds/labs showed cure.
• Patient satisfaction for pharmacist-care was high and some HIV risk behaviors improved.

## 1 Introduction

HCV is a common and highly treatable bloodborne infection that continues to lead to significant morbidity and mortality (Hofmeister et al., 2019). There is a global call to eliminate HCV, and advances in treatment—including direct-acting antivirals (DAAs)—make that possible (Grebely et al., 2021; World Health Organization, 2016). Yet efforts to reduce the burden of HCV continue to fall short in the U.S. and worldwide, especially for priority populations like people who inject drugs (PWID) (Teshale et al., 2022). PWID have documented disparities across the HCV care continuum, such that even with improvements in DAA availability, less than a quarter of PWID with known HCV are able to access treatment and even fewer reach cure status, namely “sustained virologic response” defined as undetectable HCV viral load 12 weeks after treatment (i.e., SVR12) (Corcorran et al., 2021; Day et al., 2019; Tsui et al., 2019). Within this context of low rates of treatment among PWID, in the U.S. the incidence of acute hepatitis C has more than doubled in 2020 compared to 2013 (Centers for Disease Control and Prevention [CDC], 2020).

To augment HCV elimination efforts, many regions have leveraged policy changes that aim to increase accessibility and affordability of HCV treatment. In 2018, Washington State launched a statewide initiative to eliminate HCV, including measures that negotiated discounted and fixed costs for DAAs (Auty et al., 2021; Centers for Medicare & Medicaid Services, 2019; Washington State Health Care Authority, 2020). It offers unrestricted access to HCV medications for Medicaid patients regardless of stage of fibrosis or substance and does not restrict prescribing to specialists. While these structural changes created opportunities to expand HCV treatment, Washington State has yet to realize its goals for HCV elimination, (Auty et al., 2022; Auty et al., 2021; Sulkowski et al., 2021) suggesting that more innovative models of HCV care delivery are needed (Grebely et al., 2021). A national study of Medicaid data showed that removing coverage restrictions is critical but still limited in impact without further work to expand access to DAAs (Herink et al., 2021).

Washington State has also adopted policies that expand the scope of practice for non-traditional providers like pharmacists through collaborative practice agreements (Adams and Weaver, 2016). Collaborative practice agreements that allow pharmacists to perform specific patient care functions through formal agreements with a licensed provider (CDC, 2013). Models of pharmacist collaborative care can range from co-located services in traditional healthcare settings to service delivery based in community pharmacies or other community settings. Multiple studies have demonstrated the benefit of pharmacist-led care, such as improving access to medications for opioid overdose (Akers et al., 2017; Green et al., 2015; Pollini et al., 2022; Wu et al., 2022) and HIV prevention (Gonzalez et al., 2021; Khosropour et al., 2020; Tung et al., 2018). Pharmacists have been an integral component of the U.S. Department of Veterans Affairs’ (VA) effort to eliminate HCV, and prior research (Downes et al., 2022; Hunt et al., 2022) has described pharmacist-led programs in safety-net hospitals and federally qualified health centers. A recent study conducted in Scotland demonstrated effectiveness of pharmacist-led HCV treatment among patients receiving methadone at community-based pharmacies (Radley et al., 2020). Yet, the use of pharmacists to identify and treat PWID living with HCV directly in community-based settings in the U.S. is relatively unexplored.

In the context of expanded opportunities for HCV treatment in Washington State, we developed a Pharmacist, Physician, and Patient Navigator Collaborative Care Model (PPP-CCM) for delivery of HCV treatment (Austin et al., 2023). Here we describe the results of a feasibility pilot study of the PPP-CCM model to describe clinical outcomes related to HCV treatment, as well as patient satisfaction with treatment and HIV drug and sexual risk behaviors before and after treatment.

## 2 Methods

### 2.1 Study design

This was a single-arm, prospective, observational pilot study of the PPP-CCM offered to 40 PWID who were recruited at community sites and had previously screened positive for HCV.

### 2.2 Setting/sample

Participants were recruited from community sites that serve PWID in the greater Seattle area: a non-profit organization focused on hepatitis education and health of PWID with a syringe services program (SSP), an opioid treatment program (OTP), and several emergency housing units. Potential participants were identified and referred by local providers/champions (predominantly nurses who provided care within those sites) within their respective site organizations or self-referred after seeing information in flyers/posters placed at study sites. Eligibility criteria included: (1) age ≥18 years; (2) current injection drug use (defined as injection of any drug within the past 90 days); (3) a documented positive HCV test (screening antibody or viral load); (4) not currently taking medications to treat HCV, and never previously treated with DAAs for HCV; and (5) willingness to undergo evaluation for HCV through the PPP-CCM. Participants were ineligible if they: (1) had plans to leave the Seattle area within 6 months; (2) reported impending incarceration that would disrupt clinical care; (3) were unable to speak or read in English; (4) were known to be pregnant; or (5) did not wish to be treated for HCV. The study received approval from the University of Washington Human Subjects Division and was registered on clinicaltrials.gov (NCT04698629).

### 2.3 Intervention

The PPP-CCM program was modeled after a successful pharmacist-led program for HIV pre-exposure prophylaxis (PrEP) (Tung et al., 2018) and shaped by formative discussion with stakeholders. This included qualitative research (i.e., interviews) with PWID living with HCV to assess prior experiences with and preferences for HCV treatment (Tsui et al., 2021). The formative work and implementation of PPP-CCM was described in a prior publication where full details of the model and program can be found (Austin et al., 2023). In brief, The PPP-CCM care team was comprised of two community pharmacists, two physicians (one infectious disease and the other addiction medicine trained), and a patient navigator. The Washington State Department of Health's Pharmacy Quality Assurance Commission approved a collaborative practice agreement between the pharmacists and physicians that enabled the pharmacists to provide the full spectrum of HCV care including diagnosis, counseling/education, work-up (including laboratory tests, fibrosis staging, and immunizations for hepatitis A and B), treatment, and post-treatment assessment for SVR12. The pharmacists allocated 15 h of education—which included online HCV trainings (University of Washington, 2023) and Project ECHO (Extension for Community Healthcare Outcomes) led by infectious disease and hepatology specialists (Arora et al., 2011; Scott et al., 2012)—received additional training and licensure to perform phlebotomy, and met weekly to discuss patient cases. Protocols were designed to enable pharmacists to treat patients who were not medically complex: under the existing collaborative practice agreement, patients were excluded if they (1) were living with HIV, (2) had advanced/decompensated cirrhosis (i.e., Child-Turcotte-Pugh Class B or C), (3) had previously been treated with DAAs, (4) were living with end-stage renal disease or another severe/uncontrolled comorbidity deemed to be an imminent risk to the patient's health, or (5) did not have an insurance carrier that allowed for billing of pharmacist-delivered care. Assessment for cirrhosis was generally through noninvasive indirect measures of fibrosis (APRI and FIB4) (Chou and Wasson, 2013; Lin et al., 2011; Sterling et al., 2006); however, if needed transient elastography could be obtained through referral. The PPP-CCM patient navigator was trained to provide medical case management for patients with HCV. Case management services included: (1) education around HCV diagnosis and treatment; (2) assessment of reinfection risk and access to harm reduction counseling and services; (3) resources referrals (such as for insurance, housing, employment, etc.); (3) assistance with appointment scheduling and reminders; and (4) medication management, including assistance with medication storage and assessment of side effects and adherence. The pharmacists and patient navigators were present on-site and available to engage with patients on a “drop-in” basis at the non-profit/SSP and OTP sites, and through referral at the emergency housing units.

### 2.4 Study procedures and measures

**Fig. 1:**
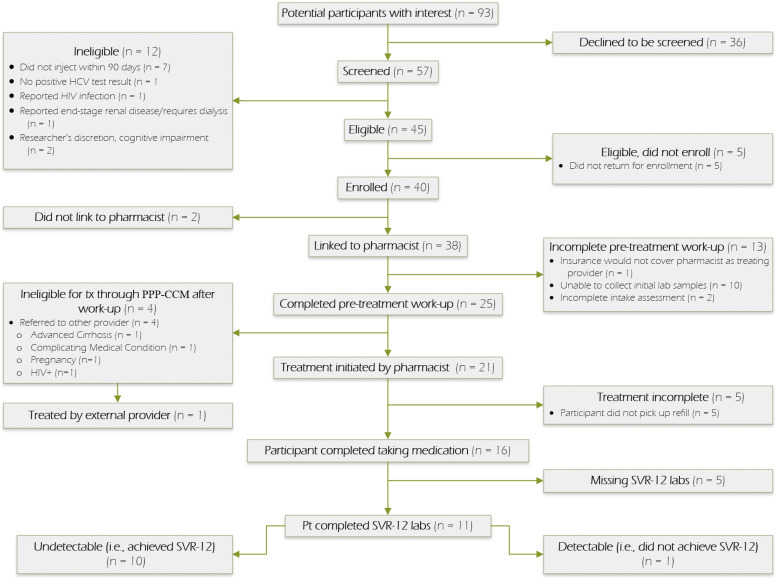
Participant flow diagram for a single-arm, prospective, observational pilot study of a community-pharmacist led HCV treatment program for people who inject drugs, Seattle, Washington.

Trained research staff conducted screenings and enrollments, obtained informed consent, and administered study questionnaires at the two study visits that occurred at baseline and six months post-study start date. Participants received a $30 reimbursement for the baseline visit and $50 for the completion of the six-month follow-up visit. After the baseline visit, participants were linked to a patient navigator to facilitate their connection to the pharmacist for HCV evaluation (Fig. 1). Baseline assessments included measures of socio-demographics; lifetime and recent (past 30-day) substance use (modified Addiction Severity Index) (McLellan et al., 1980); depressive symptoms (Patient Health Questionnaire-9, i.e. PHQ-9) (Kroenke et al., 2001); medications; HIV risk behaviors (modified HIV Risk-taking Behavior Survey) (Needle et al., 1995; Weatherby et al., 1994); questions related to healthcare utilization for HCV; and use of medications for opioid use disorder (buprenorphine and/or methadone) and overdose (current ownership of naloxone). In addition to the above information, follow-up surveys included questions developed by the researchers about HCV-related care and satisfaction with care delivery received since the baseline visit. The surveys were administered by the research staff and data were collected electronically via REDCap data collection tools (Harris et al., 2019; Harris et al., 2009). The electronic medical record (EMR) used by the pharmacists was reviewed by study staff and abstracted for information on work-up (laboratory tests, imaging, referrals, etc.), co-morbidities, receipt of DAAs, and outcomes such as treatment completion and cure. Use of the EMR was included within study activities approved by the institutional review board, and patients signed for release of information. Data were extracted from the EMR up until 1 year after the last participant was treated to assess SVR12 labs for determination of cure.

### 2.5 Study outcomes

The primary outcome was linkage with the program pharmacist for initial evaluation, which consisted of a comprehensive history taking and assessment of the patient, including labs that were indicated. We considered our primary outcome to be a measure of participant acceptability and feasibility of the intervention, as it required both patient engagement and successful coordination of care on the part of the patient navigator and pharmacist. The secondary outcomes were (1) DAA treatment initiation defined as documentation through the EMR via pharmacists’ notes that medications were dispensed to the patient; (2) DAA completion defined as documentation through the EMR via pharmacists’ notes of patient self-report of having taken all of their doses of DAA medication and having evidence of appropriate refills; and (3) SVR12/cure defined as having an undetectable HCV RNA viral load measured at least 12 weeks after treatment completion per EMR.

### 2.5 Statistical analysis

Descriptive statistics were calculated for baseline participant demographics and treatment characteristics, including age, gender, race/ethnicity, substance use in the past 30 days, HIV risk behaviors, history/experiences of seeking HCV treatment, and use of medications for opioid use disorder and overdose. The pre-specified primary feasibility outcome for this study was proportion of participants who were evaluated for HCV treatment in the community-pharmacy program. Secondary outcomes were HCV treatment initiation, completion, and SVR12/cure. Additional secondary analyses were performed to 1) compare the differences in substance use and HIV risk behaviors between baseline and follow-up and 2) assess satisfaction with pharmacist and patient-navigator care. Comparisons were performed among participants who had data from both baseline and follow-up visits using McNemar's exact test for categorical variables and Wilcoxon signed-rank test for difference in medians. Statistical tests were conducted with Stata statistical software (16.1, StataCorp LLC, College Station, TX). Analyses are reported with 95 % confidence intervals and 2-sided tests of the null hypothesis at a significance level of 0.05.

## 3 Results

Between November 2020 and October 2021, 45 PWID were screened and found to be eligible. Of those, 40 participants agreed to be in the study, consented, and enrolled. Among participants, the mean age was 43.6 years, 12 (30 %) were women, 20 (50 %) were non-white, and 15 (38 %) were unhoused (Table 1). At baseline, 80 % reported heroin use in the past 30 days and 68 % reported methamphetamine use in the past 30 days. At baseline, 15 (38 %) reported sharing injecting equipment in the past 30 days, and 18 (45 %) reported having sex without a condom in the past 30 days.

**Table 1:** Baseline demographics and treatment characteristics of participants (*N=*40).

Baseline Characteristics	n (%)^a^
Age (years), mean (SD), range	43.6 (10.3), 24–67
Gender identity	
Man	24 (60 %)
Woman	12 (30 %)
Non-binary or Transgender man or woman	4 (10 %)
Sexual orientation^b^	
Gay or Queer	3 (8 %)
Straight/heterosexual	33 (85 %)
Bisexual	3 (8 %)
Race	
Black or African-American	6 (15 %)
Native American or Alaska Native	3 (8 %)
Native Hawaiian or Pacific Islander	2 (5 %)
White	20 (50 %)
Multi-racial	5 (12 %)
Other^c^	4 (10 %)
Hispanic/Latinx ethnicity	9 (22 %)
Unhoused/housing instability in past 90 days^d^	15 (38 %)
Employment status	
Working full-time/part-time	1 (2 %)
Unemployed/Retired/Disabled	38 (95 %)
Other: sex worker	1 (2 %)
Health insurance/healthcare coverage	
None	2 (5 %)
Medicaid	38 (95 %)
Substance use in the past 30 days^f^	
Heroin	32 (80 %)
Methamphetamines/amphetamines	27 (68 %)
Fentanyl	6 (15 %)
Other non-Fentanyl pharmaceutical opioids^e^	5 (12 %)
Cocaine	8 (20 %)
Cannabis	20 (50 %)
Hallucinogens	4 (10 %)
Benzodiazepines/sedatives^b^	7 (18 %)
Any alcohol/heavy drinking^b^	17 (42 %)
None	0 %
Any injection drug us in the past 30 days?	
Yes	39 (98 %)
No	1 (2 %)
Past 30-day injection drug use^f^	
Heroin alone	20 (50 %)
Methamphetamine alone	10 (25 %)
Both heroin and methamphetamine alone	9 (22 %)
Speedballs (heroin+cocaine)	2 (5 %)
Goofballs (heroin+methamphetamine)	15 (38 %)
None	0 %
Patient Health Questionnaire (PHQ-9), major depression (score ≥10)	23 (59 %)
Prescribed OUD medications (methadone or buprenorphine) in past 12 months	
Yes	13 (32 %)
No	27 (68 %)
Currently have naloxone	
Yes	32 (80 %)
No	8 (20 %)
Any sharing of injection equipment in the past 30 days^b^	
Yes	15 (38 %)
No	24 (62 %)
Past 30-day use of shared injecting equipment (# days injecting), median (IQR)^b^	0 (0–4)
HIV-positive sex partners in past 30 days	
Yes	0 (0 %)
No	38 (95 %)
Don't know/not sure	2 (5 %)
Any episodes of condomless sex^g^ in the past 30 days	
Yes	18 (45 %)
No	22 (55 %)
Past 30-day episodes condomless sex^g^ (days), median (IQR)	0 (0–2.5)

Abbreviations: Pre-Exposure Prophylaxis (PrEP), Inter-quartile Range (IQR).

a Used scientific rounding for percentages.

b Denominator is *N=*39 due to missing data.

c Other includes: “human race,” Hispanic, and Mexican.

d Was not living in own home/apartment or home(s) of friend/family member(s) in the past 90 days.

e Includes opioids/narcotics/painkillers (Oxycontin, morphine, Darvon, Vicodin, Percocet, Demerol, Dilaudid).

f Could select more than one response option.

g Refers to vaginal or anal sex.

Twenty-one of the 40 enrolled (52 %) participants reported having not previously sought HCV treatment, which was broadly defined as having at least one encounter with a case manager, social worker, doctor, nurse, nurse practitioner, or physician assistant to request linkage to or delivery of HCV treatments (Table 2). Among the 18 who had previously sought HCV treatment, the most common location was through a primary care provider or clinic (33 %).

**Table 2:** Self-reported history of seeking HCV treatment at baseline among participants (*N=*40).

Characteristics	N (%)
Prior history of seeking HCV treatment	
Yes	18 (45 %)
No	21 (52 %)
Don't know/Not sure	1 (2 %)
What has prevented you from seeking HCV treatment in the past? (*N=*21, responded “no” to seeking HCV treatment)^a^	
Didn't know where to go	3 (14 %)
Didn't think there was any reason to / Didn't feel sick	8 (38 %)
Uncomfortable talking to a doctor	1 (5 %)
Thought I had to get sober first	4 (19 %)
Not aware of hepatitis C infection for very long	4 (19 %)
Other^b^	3 (14 %)
Where did you last seek HCV treatment? (*N=*18, responded “yes” to seeking HCV treatment)	
Primary care provider/clinic	6 (33 %)
Specialist provider/specialty clinic	1 (6 %)
Opioid treatment program or syringe service program	3 (17 %)
Community-based program	4 (22 %)
Other^c^	4 (22 %)

a Select all that apply.

b Other includes: “depressed/depression” and “saw wife's interferon treatment”.

c Other includes: Hospital (in-patient), Indian Health Board, Prison.

**Fig. 2:**
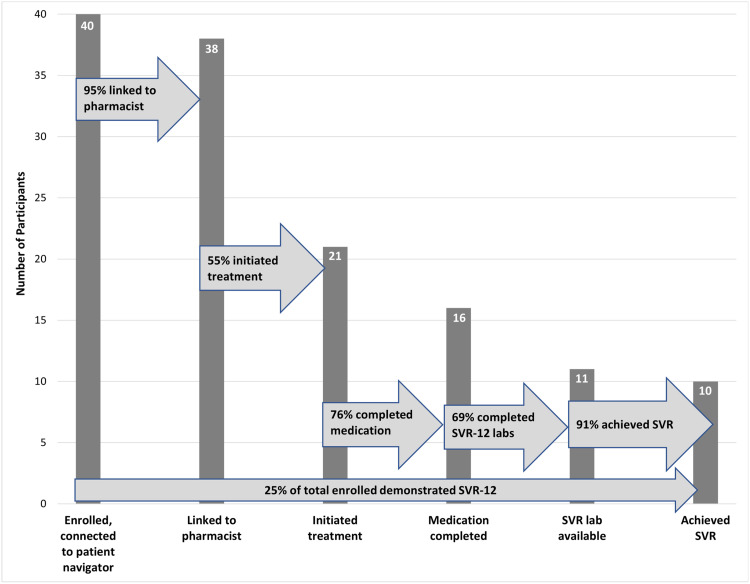
Steps of program linkage, DAA initiation, completion, and SVR12 among people who inject drugs enrolled in a community-pharmacist led HCV treatment program, Seattle, Washington (*N=*40).

Among the 40 enrolled participants, 38 (95 %; 95 % confidence interval (CI) 83–99 %) were successfully linked to the pharmacist for initial evaluation. Of those, 21/38 (55 %; 95 % CI 38–71 %) received DAA medication, and, of those, 16/21 (76 %; 95 % CI 53–92 %) completed treatment. Participants were treated with glecaprevir-pibrentasvir (20/21; 95 %) and sofosbuvir-velpatasvir (1/21; 5 %), and 16/21 (76 %) started treatment within 6 months of baseline visit. Reasons for participants not being able to initiate DAA treatment with the pharmacist are shown in Fig. 1. Among those who received and completed DAAs, 11/21 (52 %) had viral load data available. Of those with lab data, 10/11 (91 %) were found to have achieved cure (Fig. 2).

Among the 26 participants who completed both baseline and six-month study questionnaires, self-reported injecting and sexual risk behaviors were compared (Table 3). The proportion that reported any use of heroin in the past 30 days significantly decreased from 77 % at baseline to 46 % at follow-up (p-value=0.022). In contrast, fentanyl use in the past 30 days significantly increased from 15 % at baseline to 50 % at follow-up (p-value=0.004). The proportion of participants who reported injecting drugs within the past 30 days decreased significantly between baseline and follow-up visit as did the proportion reporting having sex without a condom in the past 30 days.

**Table 3:** Comparisons of past 30-day substance use and HIV risk behaviors between baseline and 6-months among participants with follow-up visit data (*N=*26).

	Participants		
Characteristics, n (%)	Baseline, not lost to follow-up	6-month follow-up	P-value^a^
Substance use in past 30 days			
Heroin	20 (77 %)	12 (46 %)	0.022
Methamphetamines/amphetamines	16 (62 %)	17 (65 %)	1.00
Fentanyl	4 (15 %)	13 (50 %)	0.004
Other non-Fentanyl pharmaceutical opioids prescription^b^	0 (0 %)	0 (0 %)	NA
Cocaine	5 (19 %)	5 (19 %)	1.00
Cannabis	15 (58 %)	15 (58 %)	1.00
Hallucinogens	2 (8 %)	2 (8 %)	1.00
Benzodiazepines/sedatives	4 (16 %)^c^	5 (19 %)	1.00
Any alcohol/heavy drinking	12 (46 %)	13 (50 %)	1.00
Any injection drug use in the past 30 days			
Yes	26 (100 %)	19 (73 %)	0.016
No	0 (0 %)	7 (27 %)	
Past 30-day injection drug use (# days injecting), median (IQR)	3 (2–5)	1 (0–4)	0.134
Past 30-day injection drug use^d^			
Heroin alone	13 (50 %)	7 (27 %)	0.070
Methamphetamine alone	8 (31 %)	7 (27 %)	1.00
Both heroin and methamphetamine alone	5 (19 %)	3 (12 %)	0.625
Speedballs (heroin+cocaine)	1 (4 %)	2 (8 %)	1.00
Goofballs (heroin+methamphetamine)	9 (35 %)	5 (19 %)	0.125
Any sharing of injection equipment in the past 30 days			
Yes	10 (38 %)	7 (27 %)	0.508
No	16 (62 %)	19 (73 %)	
Past 30-day use of shared injecting equipment (# days sharing), median (IQR)	0 (0–2)	0 (0–1)	0.581
Any episodes of condomless sex^e^ in the past 30 days			
Yes	11 (42 %)	4 (15 %)	0.016
No	15 (58 %)	22 (85 %)	
Past 30-day episodes of condomless sex^e^ (# days with condomless sex), median (IQR)	0 (0–2)	0 (0–0)	0.065

a McNemar's exact test for categorical variables. Wilcoxon signed-rank test for difference in medians. Tests are among the 26 participants with both baseline and follow-up data available.

b Includes opioids/narcotics/painkillers (Oxycontin, morphine, Darvon,Vicodin, Percocet, Demerol, Dilaudid).

c Denominator for benzodiazepines/sedatives is *N=*25 due to one missing observation.

d Could select more than one response option.

e Refers to vaginal or anal sex.

There was a high degree of satisfaction with care delivered by the patient navigator and pharmacist based on subsamples of participants who completed the relevant questions at the 6-month survey (Supplemental Figs. 1 and 2). Among the 10 participants who had initiated HCV treatment and answered questions about the pharmacist's care, 100 % responded “agree” or “strongly agree” to statements that they had a positive experience with the pharmacist, felt the pharmacist was non-judgmental, and would refer other PWID to the pharmacists for treatment.

## 4 Discussion

This study evaluated the feasibility and acceptability of the PPP-CCM program for HCV care among PWID. Nearly all participants enrolled were successfully linked to a pharmacist for evaluation, and more than half were started on DAAs. Of those with final lab results, 91 % were shown to have achieved cure—a finding that is notable in the context of active substance and injection drug use. Additionally, participants treated in the program expressed a high degree of satisfaction with the care provided by the pharmacist and patient navigator and demonstrated improvements in injecting and sexual risk behaviors at follow-up visit. These results provide preliminary evidence that a model of pharmacist-led treatment embedded in community sites can be successful in providing care leading to cure and other health benefits among PWID living with HCV.

Our results add to the body of literature demonstrating that pharmacists can expand access to essential medications for persons who use drugs and persons living with HCV. Prior research has shown that pharmacists can play a key role in the provision of medications for prevention/treatment of HIV, overdose, and OUD (Akers et al., 2017; Green et al., 2015; Khosropour et al., 2020; Tung et al., 2018; Wu et al., 2022). Pharmacists have also been an integral component of HCV care at the VA healthcare system, contributing to successful elimination efforts (Yang et al., 2017) across the VA system in the U.S. It is estimated that nearly one third of DAA treatment in the VA has been managed by clinical pharmacists with similar cure rates to specialists (Gonzalez et al., 2021). In the U.S. outside the VA system, recent research has described pharmacist-led programs in a safety-net hospital and federally qualified healthcare system (Koren et al., 2019; Olea et al., 2018). In Scotland, offering HCV treatment through pharmacists for persons receiving methadone through community pharmacies has demonstrated significantly higher odds of HCV cure as compared to treatment as usual (Radley et al., 2020). To our knowledge, ours is the first study in the U.S. to demonstrate feasibility of expanding pharmacist-led care to community sites for HCV treatment to PWID. Our intervention was also unique in that protocols allowed pharmacists to provide the full range of HCV care, from testing to diagnosing, offering treatment, and assessing for cure. Thus, learnings from this study suggest that pharmacists can expand access to HCV care to a priority population (PWID), and achieve high rates of cure, similar to other studies of non-specialist providers (Kattakuzhy et al., 2017; Lasser et al., 2017; Ngo et al., 2021).

Our pilot study demonstrates the ability of a new care delivery model to provide HCV evaluation and treatment for PWID. Prior work has repeatedly demonstrated that PWID face many barriers to accessing treatment in traditional formats, including rigid delivery structures, lack of specialty providers, and stigmatizing interactions with healthcare providers (Artenie et al., 2015; Asher et al., 2016; Austin et al., 2022). As shown in our prior work, (Austin et al., 2022; Tsui et al., 2021) concerns about stigma and requirements for abstinence prevent PWID from seeking care for HCV from healthcare systems. Thus, there is a need to deliver HCV treatment providers to community sites where PWID often seek care, such as addiction and harm reduction programs (Alavi et al., 2013; Butner et al., 2017; Tsui et al., 2019), which pharmacists may be well-positioned to address. Such task-shifting and decentralization of HCV care away from traditional care settings to community sites will be essential to achieving HCV elimination (Oru et al., 2021). On average, 38 % of our participants were experiencing unstable housing, and 68 % were not actively engaged in MOUD. Despite these obstacles, more than half of participants initiated HCV medications and most of those receiving medications completed and were cured, demonstrating the potential of this model to reduce barriers for harder to reach populations. Expansion of this program may contribute to achieving WA State elimination goals and help achieve equity in treatment access. HCV treatment uptake among PWID in WA State remains low, particularly for young adults, women and persons who are unhoused (Corcorran et al., 2021).

The PPP-CCM pilot did not deliver treatment to all participants. Failures to initiate treatment often stemmed from inability of staff to obtain blood samples: there were 10 patients who had uncompleted labs, and four of those who completed treatment lacked confirmation of cure with SVR12 test results. Efforts to obtain blood samples were substantial: there were numerous individuals who were trained in phlebotomy at the site and if one person was unsuccessful, they would ask another individual to try. When on-site phlebotomy was unsuccessful, the patient would be instructed to come back another day for another attempt. These results highlight that phlebotomy requirements remain a substantial structural healthcare barrier to HCV treatment/cure in the DAA era that must be overcome for PWID, perhaps through point-of-care HCV RNA diagnostics (Madden et al., 2018). Furthermore, there were patients who were lost to follow-up in all phases (pre-, during, and post-treatment), which speaks to challenges even with the use of patient navigators. Thus, this work highlights that may not be any single care model that will work for all individuals and multiple strategies/pathways are needed for PWID. Although we considered analyses to examine for differences among those lost to follow-up, it was felt that given the small sample it would be not be advisable since differences that happen by chance will have large impact. Approximately one-quarter of participants did not complete treatment, which is similar to another large study of HCV treatment models in PWID (Litwin et al., 2022). None of the participants who did not complete DAAs had SVR12 labs drawn to establish whether they were cured, yet 40 % took at least half their medication doses. There is emerging evidence demonstrating that cures can occur even with suboptimal adherence: a recent study showed that patients who achieved at least 50 % adherence resulted in an overall SVR rate of 99 % (Norton et al., 2020). Future research should explore how the model can be improved upon, including offering less invasive point-of-care testing and strategies to maintain engagement, as well as the feasibility of expanding to other pharmacies and settings, including rural areas.

Follow-up surveys conducted 6 months after enrollment from this study demonstrated some positive changes in behaviors between baseline and follow-up surveys, as there were significant reductions in the percentage reporting injecting drugs and engaging in condomless sex in the past 30 days. These results should be interpreted cautiously as there was no comparison group in the study. Also, the percentage reporting use of heroin declined, however, these changes may reflect regional patterns of drug use and drug supply, most notably the shift from heroin to fentanyl (O'Donnell et al., 2021). This study launched recruitment early in the COVID-19 pandemic, which coincided with the emergence of fentanyl in drug supplies on the West coast of the U.S., including the Seattle area (Palamar et al., 2022; Reinhart et al., 2022). It should also be noted that over 60 % of our participants reported regular methamphetamine use, both in tandem with heroin use and use alone, which is consistent with prior local research (Glick et al., 2018). Given that methamphetamine has been shown to be a risk factor for failure to reach HCV cure or increased risk of HCV reinfection (Litwin et al., 2022), more work is needed to understand how to better address the complex patterns of drug use that further complicate PWID efforts to treat HCV.

There are a number of study limitations. The learnings from this study reflect a pilot study that is limited in scope and sample size and therefore findings should be viewed cautiously. Our pilot was implemented in Washington, a state that offers pharmacists a broader scope of practice than many other states, and one that allows pharmacists to bill for their services providing clinical care. Such facilitators and barriers to model implementation have been previously described (Austin et al., 2023). Learnings from this work may not translate to other states where pharmacist roles are more restricted. However, the COVID-19 pandemic has demonstrated that pharmacists can be frontline caregivers in the context of a public health emergency: HCV is also a public health crisis that has recently been deemed a national priority by the current presidential administration (Fleurence and Collins, 2023). Additionally, Washington State offers unrestricted access to HCV medications for Medicaid patients—i.e., there are no limits based on stage of fibrosis or substance use nor requirements for prior authorization or specialist prescriber—which may not be the case in all states, although many are adopting similar policies over time (Center for Health Law and Policy Innovation of Harvard Law School, 2023). Nevertheless, Washington continues to fall behind treatment goals for HCV elimination (Auty et al., 2021; Florko, 2022), and thus the learnings from the pilot may provide a valuable new strategy for expanding the pool of treaters within the state. A limitation of the study is the lack of SVR12 data on all participants who were treated in the study. However, our rates of SVR12 lab completion are comparable to other “real-world” studies of PWID (Litwin et al., 2022) and reflects the population which was actively using substances and not engaged in primary care or addiction treatment. Finally, the pharmacy that we partnered with for this study is a community-based pharmacy with a long-standing interest/commitment to providing care for vulnerable populations such as people who use drugs; we acknowledge that not all pharmacies/pharmacists will be similarly inclined. Nevertheless, this pilot is an important first step that could encourage other pharmacies to offer similar programs and/or policymakers to devise ways to support and incentivize such programs. There is a need for future research to ascertain the willingness of pharmacists and physicians to utilize this model, and to assess how barriers to adoption might be addressed.

In summary, this study demonstrated the feasibility of a community-based pharmacist-led program that provided HCV care including treatment leading to cure for PWID. Learnings from this study showcase how utilizing pharmacists as HCV care providers, in partnership with patient navigators and physicians, directly in the community may reduce barriers to access and receipt of HCV treatment for PWID.

## CRediT authorship contribution statement

**J.I. Tsui:** Conceptualization, Methodology, Investigation, Writing – original draft, Writing – review & editing, Supervision, Project administration, Funding acquisition. **A.J. Gojic:** Investigation, Data curation, Project administration, Writing – review & editing. **K.A. Pierce:** Investigation, Project administration, Writing – review & editing. **E.L. Tung:** Conceptualization, Investigation, Project administration, Supervision, Writing – review & editing. **N.C. Connolly:** Investigation, Project administration, Writing – review & editing. **A.C. Radick:** Formal analysis, Data curation, Writing – review & editing, Project administration. **R.R. Hunt:** Investigation, Data curation, Writing – review & editing. **R. Sandvold:** Investigation, Project administration, Writing – review & editing. **K. Taber:** Investigation, Project administration, Writing – review & editing. **M. Ninburg:** Conceptualization, Investigation, Supervision, Writing – review & editing. **R.H. Kubiniec:** Investigation, Project administration, Writing – review & editing. **J.D. Scott:** Methodology, Investigation, Writing – review & editing, Funding acquisition. **R.N. Hansen:** Methodology, Investigation, Writing – review & editing, Funding acquisition. **J.D. Stekler:** Conceptualization, Methodology, Writing – review & editing, Funding acquisition. **E.J. Austin:** Methodology, Investigation, Writing – review & editing, Project administration. **E.C. Williams:** Conceptualization, Methodology, Investigation, Supervision, Writing – review & editing, Funding acquisition. **S.N. Glick:** Conceptualization, Methodology, Formal analysis, Writing – review & editing, Supervision, Funding acquisition.

## Declaration of Competing Interest

The authors declare that all procedures were performed in compliance with relevant laws and institutional guidelines. Appropriate ethical safeguards, including privacy rights of human participants, and ethical approvals were obtained through the University of Washington's Institutional Review Board.

## Appendix Supplementary materials

Image, application 1
