# Supplementary material for: Pilot study of a community pharmacist led program to treat hepatitis C virus among people who inject drugs
Source: Drug Alcohol Depend Rep. 2023 Dec 23;10:100213. doi: 10.1016/j.dadr.2023.100213 (PMC10796962; doi:10.1016/j.dadr.2023.100213)
Supplement: Supplementary file 1 [file mmc1.docx]

**Supplementary Material**

**Supplemental Figure 1.** Likert scale survey responses to questions regarding satisfaction with patient navigators among PWID respondents who met with a patient navigator to start HCV treatment, Seattle, WA (N=18)

**Supplemental Figure 2.** Likert scale survey responses to questions regarding satisfaction with pharmacist care among PWID respondents who initiated HCV treatment with the study pharmacist, Seattle, WA (N=10)
